# Salivary levels of inflammatory and anti-inflammatory biomarkers in periodontitis patients with and without acute myocardial infarction: implications for cardiovascular risk assessment

**DOI:** 10.3389/froh.2024.1332980

**Published:** 2024-02-16

**Authors:** Sudhir Varma, Biju Thomas, K. Subrahmanyam, Kimberly Duarte, Mohammed A. Alsaegh, Divya Gopinath, Sam T. Kuriadom, Jayaraj Narayanan, Vijay B. Desai, Al Moutassem B. Khair, Kelvin I. Afrashtehfar

**Affiliations:** ^1^ Department of Clinical Sciences, Ajman University, Ajman, United Arab Emirates; ^2^ AB Shetty Memorial Institute of Dental Sciences, Mangalore, India; ^3^ NITTE (Deemed to be University), Mangalore, India; ^4^ K.S. Hegde Hospital, Mangalore, India; ^5^ College of Dental Medicine, University of Sharjah, Sharjah, United Arab Emirates; ^6^ Department of Basic Sciences, Ajman University, Ajman, United Arab Emirates; ^7^ Bern Center for Precision Medicine, Medical School, University of Bern, Bern, Switzerland; ^8^ Department of Orthodontics, University Hospital RWTH Aachen, Aachen, Germany

**Keywords:** cardiovascular diseases, salivary biomarkers, myocardial infarction, adiponectin, C-reactive protein, chemokine CCL3, macrophage inflammatory proteins, periodontitis

## Abstract

**Background:**

Periodontitis is initiated by a dysbiotic activity and furthermore leads to a chronic inflammatory response. The presence of pro-inflammatory markers plays an important role in the inflammatory load. Macrophage inflammatory protein-1 alpha (MIP-1α) and C-reactive protein (CRP) are pro- inflammatory biomarkers that quantify clinical and subclinical inflammation in cardiac ischemia in cardiac inflammation and disease. Adiponectin is an anti-inflammatory marker associated with good health. The susceptibility of periodontitis patients to cardiovascular events needs to be evaluated.

**Objective:**

This study aims to assess the levels of biomarkers in periodontitis patients with and without acute myocardial infarction (AMI) compared to controls.

**Material and methods:**

Pro-inflammatory and anti-inflammatory analytes were examined by collecting unstimulated saliva from three groups (*n* = 20/each): healthy individuals, individuals with stage III periodontitis, and post-myocardial infarction patients with stage III periodontitis. The samples were collected within 48 h of AMI.

**Results:**

Adiponectin levels were significantly lower in patients with periodontitis with and without AMI compared to controls, while CRP and MIP-1α were significantly higher in patients with periodontitis with and without AMI compared to controls. The highest titers for MIP-1α and CRP were detected among patients with periodontitis with and AMI.

**Conclusion:**

Our study provides possible evidence of the association between periodontitis and salivary analytes that occur in tandem with cardiovascular disease. The lower levels of Adiponectin and higher levels of CRP and MIP-1α in patients with periodontitis indicate that this condition is a potential risk factor for cardiovascular disease. The findings emphasize the importance of early detection and intervention for periodontitis patients to prevent cardiovascular events.

## 1 Background

Periodontitis is a chronic inflammatory disease caused by various periodontopathic microorganisms that invade the immunoregulatory mechanisms of the body. Several studies have reported a link between periodontal and cardiovascular diseases (1, 2). Periodontitis can increase the risk for heart disorders due to several biologically mediated mechanisms that may enhance the inflammatory response in atheromatous plaques due to periodontal infection (3). This includes the production of higher levels of systemic mediators of inflammation stimulated by bacteria and their products in sites distant to the oral cavity, which creates an inflammatory and prothrombotic status (4, 5). Studies have reported an association between coronary heart disease (CHD) and periodontal disease (6, 7). However, few studies have provided conclusive evidence about cardiac outcomes concerning periodontal severity. The inconsistency of studies may be due to different definitions of exposure safety, highlighting the need for consensus on periodontal- cardiac interrelationships.

The release of cytokines that mobilize leukocytes and play an essential role in osteoclast formation orchestrates an intermediary modulatory mechanism in the destruction of periodontal connective tissue (5, 6). While various circulating molecules have been detected in the saliva of periodontal patients, chemokines have lately been accorded particular interest. Macrophage inflammatory protein-1 alpha (MIP-1α), a chemotactic chemokine secreted by macrophages, has several functions, including stem cell inhibition, recruitment of inflammatory cells, and maintaining the effector immune response. MIP-1α exhibits a pivotal role in the pathogenesis of inflammatory diseases leading to the resorption of bone (7). Adiponectin (APN), an adipokine released from adipose tissue into peripheral blood, possesses anti-inflammatory action. APN alleviates the spread of inflammation by reducing the release of inflammatory cytokines by the diseased periodontium. APN also exhibits a cardio-protective action by accumulating in the bloodstream and binding to T-cadherin, a specific receptor for the high molecular weight APN, thus protecting the heart's functions (8, 9). It plays an anti-atherogenic role in the progression and development of atherosclerosis (10, 11). C-reactive protein (CRP) is an acute-phase marker for inflammation produced in response to many forms of tissue injury and associated with periodontal diseases. CRP is elevated in cardiovascular diseases leading to inflammatory changes in the coronary vessels (12).

Early detection of these biomolecules plays an essential role in periodontal disease outcomes.

Saliva holds great promise for its diagnostic ability among body fluids, mainly due to its non- invasiveness, cost-effectiveness, and smaller aliquots (13). The high sensitivity and discriminatory role in evaluating biomarkers in periodontal disease offer a discretionary role in chairside diagnostics, thereby utilizing its translational capacity (14, 15). Various studies have investigated salivary analytes and their role in the progression of systemic diseases, mainly metabolic syndromes, diabetes mellitus, and myocardial infarction (13, 16, 17).

Studies have reported an association between CHD and periodontal disease (2, 18). Nevertheless, the studies have employed different definitions of exposure safety, providing a lack of consensus on periodontal-cardiac interrelationships. Therefore, the present study aimed to assess and compare the discriminatory levels of salivary MIP-1α, adiponectin, and C-Reactive protein in healthy individuals, stage III periodontitis patients, and post-myocardial infarction patients with stage III periodontitis.

## 2 Materials and methods

### 2.1 Study registration and ethical components

This cross-sectional clinical study involved patients visiting the Department of Periodontology, ABSMIDS and patients admitted to the Department of Cardiology, KSHEMA. Ethical clearance was granted by the Central Ethics committee-NU/CEC/2020/0281. The study was conducted in accordance with the Helsinki Declaration of 1975, revised in 2013. The study period was from April 2022 to October 2022. The study is registered on ClinicalTrials.gov-NT5314192. Informed consent was obtained from the study participants prior to saliva sample collection selected from the Department of Periodontology after explaining the study procedure. In the case of post-myocardial infarction patients, informed consent was obtained from conscious patients in the intensive care unit (ICU) or their bystanders. The collected samples were assayed in a de-identified manner to ensure the participants' privacy.

### 2.2 Selection criteria

The inclusion criteria consisted of male adult subjects who were at least 20 years old, had a minimum of 20 teeth, had no history of using anti-inflammatory medication in the last two months, a body mass index (BMI) below 25 kg/m^2^ as stated by Ebersole et al. (19), and had no history of periodontal treatment performed in the last six months. The exclusion criteria were based on patients with a history of stroke, immune diseases, taking steroid medications, suffering from organ complications/failure, mucosal inflammatory conditions, and salivary gland dysfunction.

### 2.3 Study groups distribution and characteristics

**Figure 1:**
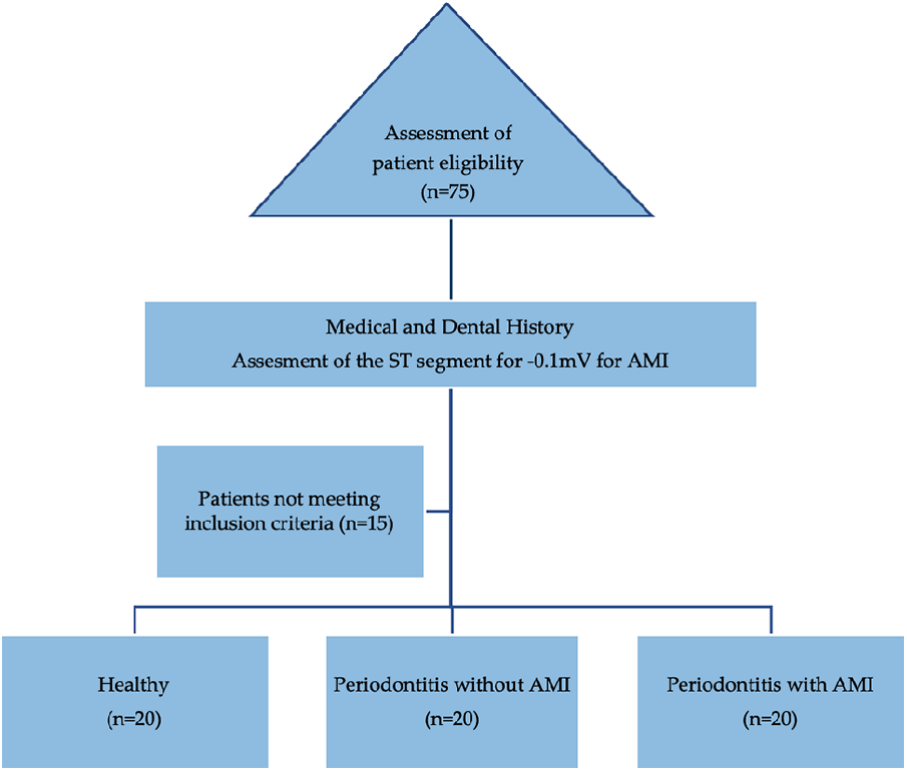
Flowchart of the recruitment of participants icluded in the cross-sectional study. ST, segment of the T-wave (on an electrocardiogram); AMI, acute myocardial infarction.

Case history of the included participants was recorded, including demographics, medical and dental status. All patients were diagnosed based on AAP classification (1). Age and gender of matched patients were used to determine the recruited three groups of 20 male patients (total, *n* = 60) (Table 1; Figure 1).

**Table 1:** Demographics in the control and test subjects.

	Control (Group 1)	Test groups		Total
		Stage III periodontitis (Group 2)	Stage III periodontitis with AMI (Group 3)	
Number of participants	20	20	20	60
Age (years)*	42.5	51.4	54	52.7
Male	Yes	Yes	Yes	
Race (ethnicity)	Asian	Asian	Asian	
Alcohol use (*n*)	6	6	8	6.6
Tobacco use (*n*)	2	5	12	6.3*
Smokeless tobacco use (*n*)	5	9	10	8*

* Mean value; *n*-Number of users.

Group 1 included healthy patients with no bleeding on probing (BOP < 10%), probing depth (PD) ≤3 mm, no clinical attachment loss (CAL), no radiographic evidence of marginal bone loss (MBL), and no sign of any inflammatory lesions in the oral mucosa.

Group 2 included stage III periodontitis patients with radiographic bone loss extending to the middle third of the root. Probing pocket depth (PPD) was measured using William's periodontal probe (Williams probe, Hu-Friedy, Chicago, IL, USA) at six sites for each tooth.

Group 3 included acute myocardial infarction (AMI) patients with stage III periodontitis. The cardiologist diagnosed acute myocardial infarction, and a periodontist registered the periodontal status in these patients at the Cardiology institute using a portable dental x-ray unit (khd11, khDental, Calgary, CA). Extension of radiographic bone loss to the middle third of the root and beyond, along with vertical bone loss, was considered. PPD and CAL were not performed to avoid the possibility of bacteremia during probing.

### 2.4 Steps and data collection

Patients diagnosed with acute ST-elevation myocardial infarction (STEMI) by the cardiologist based on the elevation of ST segments on the ECG by −0.1 mV in contiguous leads, in addition to signs and symptoms of myocardial ischemia and increased cardiac biomarkers (troponin 1 > 99th percentile of the upper reference limit, cutoff 0.04 ng/ml), were enrolled in the study. NSTEMI was diagnosed in patients with signs and symptoms of myocardial ischemia and depression in the ST-segment on the ECG, new left bundle branch block, or new *Q* wave pathology, confirmed by a positive troponin 1 test. Periodontal status was evaluated visually at the patient's bedside to avoid hindering medical management, and patients with stage III periodontitis were included.

Saliva samples were collected within 48 h of the cardiac event following the technique described by Henson and Wong (20). Participants were instructed to avoid eating, drinking, or performing oral hygiene procedures for at least one hour before saliva collection. Ten minutes before collection, subjects rinsed their mouths with tap water for approximately 30 s. Unstimulated whole expectorated saliva (5 ml) was collected between 9 am to 11 am to minimize diurnal variations using sterile polypropylene tubes. Samples were transported on ice to the Central Research Laboratory and centrifuged at 2,500 rpm for 10 min. The supernatant was aliquoted and stored at −80 °C until the assay was performed.

Levels of MIP-1 alpha (Shanghai Coon Koon Biotech Co Ltd, China), Adiponectin (ab108786, Abcam, USA), and C Reactive Protein (Salimetrics, Gen II, USA) in saliva samples were assessed using double antibody sandwich technology enzyme-linked immunosorbent assay (ELISA) following the manufacturer's instructions. For MIP-1alpha, samples and standards were added to antibody pre-coated wells, followed by the addition of conjugate reagent to form an immune complex. The color of the solution positively correlated with the concentration of MIP-1 alpha. For Adiponectin, samples and standards were bound to immobilized and biotinylated antibodies to form a sandwich complex. The color intensity of the enzymatic reaction was directly proportional to the adiponectin concentration. For C Reactive Protein, samples were placed in microwells coated with specific human CRP antibodies, and the concentrations were measured by the color change from blue to yellow.

### 2.5 Statistical analysis

A power analysis was conducted to determine the appropriate sample size for the study. Based on a.40 f- effect size and a 5% significance level under one-way ANOVA with a power of 80%, a minimum of 20 subjects per group was required to detect a statistically significant difference. However, due to challenges in identifying gender and age-matched participants, a total sample size of 75 was determined to increase the power of the study. A *post hoc* power calculation was performed for MIP-1α, and a power of 99% was observed.

The collected data were entered into a Microsoft Excel spreadsheet and analyzed using IBM SPSS Statistics, Version 22 (Armonk, NY: IBM Corp). Descriptive statistics were used to present continuous variables, including mean, median, standard deviation, and quartiles. One-way ANOVA followed by the Tukey *post hoc* test was used to compare adiponectin and MIP-1α levels between the three study groups, while the Kruskal–Wallis test followed by Mann–Whitney *U*-test, was used to compare CRP levels. A *P* value of <0.05 was considered statistically significant.

## 3 Results

The expression levels of adiponectin, MIP-1 alpha, and CRP significantly varied between MI patients with periodontitis, patients with periodontitis, and controls (Table 2). Controls showed the highest adiponectin expression (941.86 ± 76.50 ug/ml), whereas the least was observed for MIP-1 alpha (310.62 ± 32.32 pg/ml). Group 3 (MI patients with periodontitis) had the lowest adiponectin expression (750.80 ± 28.76 pg/ml), whereas the highest MIP-1 alpha expression (443.94 ± 37.74 pg/ml) was observed in this group. Group 2 had mean adiponectin and MIP-1 alpha levels of 840.27 ± 22.54 pg/ml and 366.60 ± 34.77 pg/ml, respectively (Table 2). Tukey's *post hoc* test demonstrated that all group means were significantly different from each other, and the levels of adiponectin and MIP-1 alpha significantly varied between each group in pairwise comparison (Table 3).

**Table 2:** Comparison of adiponectin and MIP 1—alpha between the study groups.

	Group	*n*	Mean	SD	Minimum	Maximum	ANOVA	
							F	*p-*value
Adiponectin (ug/ml)	1	20	941.86	76.50	882.36	1,210.50		
	2	20	840.27	22.54	801.79	871.20	76.29	<0.001
	3	20	750.80	28.76	707.75	805.86		
MIP 1—alpha (pg/ml)	1	20	310.62	32.32	270.20	376.85		
	2	20	366.60	34.77	309.90	425.60	73.11	<0.001
	3	20	443.94	37.74	356.95	486.85		

*n*, sample size; SD, standard deviation; F, Factor ratio; group 1, healthy patients; groups 2, stage III periodontitis patients; group 3, acute myocardial infarction (AMI) patients with stage III periodontitis.

**Table 3:** Pairwise comparison of adiponectin and MIP 1—alpha between the study groups.

	Groups sub-groups		Mean difference	SE	*p-*value	95% Confidence interval	
						Lower bound	Upper bound
Adiponectin (ug/ml)	1	2	101.59	15.48	<0.001*	64.35	138.84
		3	191.06	15.48	<0.001*	153.81	228.30
	2	3	89.46	15.48	<0.001*	52.22	126.71
MIP 1—alpha (pg/ml)	1	2	−55.99	11.07	<0.001*	−82.63	−29.34
		3	−133.32	11.07	<0.001*	−159.97	−106.68
	2	3	−77.34	11.07	<0.001*	−103.98	−50.69

Tukey *post hoc* test; SE, standard error.

* Statistically significant (*p* < 0.05).

CRP did not follow the normality assumption, so the Kruskal–Wallis test was used. There was a significant difference in CRP values between the three groups. Salivary CRP levels were significantly elevated in MI patients with periodontitis, followed by periodontitis patients and then controls. Mann–Whitney *U*-test demonstrated that all group means were significantly different from each other, with Group 3 having the highest CRP level of 0.50 ± 0.50 mg/dl followed by Group 2 (0.14 ± 0.15 mg/dl) and Group 1 (0.09 ± 0.02 mg/dl) having the lowest (Table 4).

**Table 4:** Comparison of CRP between the study groups.

Group	*N*	Mean (SD)	Range	Median (Q1–Q3)	Mann–Whitney *U*-test		Kruskal–Wallis test		
					Chi square value	*p*-value	1 vs. 2	1 vs. 3	2 vs. 3
1	20	0.09 (0.02)	0.06–0.13	0.08 (0.08–0.11)	22.79	<0.001*	0.02*	<0.001*	0.001*
2	20	0.14 (0.15)	0.08–0.73	0.10 (0.09–0.13)					
3	20	0.50 (0.50)	0.08–1.50	0.30 (0.12–0.90)					

Group 1, healthy patients; groups 2, stage III periodontitis patients; group 3, acute myocardial infarction (AMI) patients with stage III periodontitis.

* Statistically significant (*p *< 0.05). CRP (mg/dl).

Regarding the correlation between levels of adiponectin, MIP-1 alpha, and CRP in the study groups, although the values indicated higher MIP-1 alpha levels and CRP in inflammatory conditions, there was no significant correlation between the levels of adiponectin, MIP-1 alpha, and CRP in all three study groups (Tables 5, 6).

**Table 5:** Correlation between adiponectin and MIP 1—alpha in each study group.

Group		Adiponectin MIP 1—alpha
1	*r*	0.06
	*p*-value	0.80
2	*r*	0.04
	*p*-value	0.86
3	*r*	0.24
	*p*-value	0.31

Group 1, healthy patients; groups 2, stage III periodontitis patients; group 3, acute myocardial infarction (AMI) patients with stage III periodontitis. Adiponectin (ug/ml); MIP-1alpha (pg/ml).

**Table 6:** Correlation between adiponectin, MIP 1—alpha and CRP in each study group.

Group		Adiponectin CRP	MIP 1—alpha CRP
1	Spearman's Rho	−0.21	0.11
	*p*-value	0.39	0.65
2	Spearman's Rho	−0.06	−0.04
	*p*-value	0.80	0.88
3	Spearman's Rho	−0.03	−0.28
	*p*-value	0.90	0.24

Group 1, healthy patients; groups 2, stage III periodontitis patients; group 3, acute myocardial infarction (AMI) patients with stage III periodontitis. Adiponectin (ug/ml); MIP-1alpha ((pg/ml); CRP (mg/dl).

## 4 Discussion

This study aimed to evaluate the levels of MIP-1 alpha, adiponectin, and CRP in unstimulated whole saliva from 60 adult subjects grouped as healthy, stage 3 periodontitis, and post-AMI subjects with stage 3 periodontitis. To the best of our knowledge, no previous study has explored the role of CRP, MIP-1 alpha, and adiponectin together, despite their potential significance as pro-inflammatory and anti- inflammatory markers, respectively. Although some studies have investigated MIP-1 alpha with other cytokines in the context of health, gingivitis, and periodontitis, which are known to contribute to periodontal dysbiosis (19, 21–23), the present study sought to fill this gap in knowledge.

While some previous studies have associated poor oral hygiene with an increased risk of chronic vascular disease (24–26), we did not consider this factor in our study design to avoid limiting the inferences as the study progressed. Our results showed that CRP levels were significantly elevated in the saliva of both MI and periodontitis patients compared to healthy controls, consistent with previous research reporting elevated CRP levels in STEMI patients (27). Moreover, CRP levels were positively associated with BMI and intima-media thickness (19). Other studies have shown that serum CRP levels and fibrinogen are significantly increased in acute coronary syndrome patients, including those with periodontitis (28, 29). Although some researchers have questioned the validity of using salivary CRP as a reliable biomarker, as several factors could influence its concentration, including the number of teeth, oral hygiene, and body weight, Ebersole et al. reported elevated salivary CRP levels in MI patients with more teeth. In contrast, serum CRP levels were elevated in MI patients regardless of the number of teeth (19).

Several studies have previously validated the presence of both salivary and serum adiponectin levels in healthy volunteers, particularly those aged 40 and younger (30, 31). Adiponectin has been shown to have an anti-inflammatory effect by activating macrophages and reducing the proliferation of pro- inflammatory cytokines (32, 33). Moreover, the presence of adiponectin in non-diabetic patients has been associated with reduced severity of cardiac disease (34). Low serum adiponectin levels have been linked to the initiation of coronary heart disease progression (35). Our findings showed that salivary adiponectin levels were significantly higher in controls than in periodontitis and AMI patients, indicating a potential vascular protective role. In addition, our results showed a negative correlation between salivary CRP and adiponectin levels, supporting their potential use as risk indicators of cardiovascular disease, which is consistent with previous research (36, 37).

Interestingly, our results showed a reverse relationship between salivary adiponectin and CRP levels in controls vs. AMI patients, which contrasts with another study using serum samples, which found that Adiponectin and CRP levels were lower and higher, respectively, in both controls and MI patients, but only in those with higher BMI, which we excluded from our selection criteria (38–40). This discrepancy could be due to adipose tissue, which could modulate pro-inflammatory activity and lead to adverse CRP levels in serum. Our study showed that salivary adiponectin levels reflected the oral health status, with increased levels in healthy controls, which was not observed in AMI subjects.

The presence of C-reactive protein (CRP) levels in serum and saliva was found to be directly correlated with acute myocardial infarction (AMI) patients, consistent with previous studies (19, 29). In contrast, periodontitis and its relation to coronary artery study (PAROKRANK) found elevated clinical signs of periodontal inflammation and myeloperoxidase and MMP-8 biomarkers in non-myocardial infarction patients (41, 42).

In this study, we observed a high titer presence of macrophage inflammatory protein (MIP) 1- alpha in AMI patients, which suggests the possibility of macrophage modulation and lymphocytes initiating an inflammatory cascade. This finding contradicts another study that reported low levels of MIP-1α in periodontitis patients (43). Our study used gingival crevicular fluid (GCF) as a diagnostic utility tool. We found a positive statistical correlation between MIP-1α and CRP levels in the periodontal status of groups 2 and 3, consistent with other studies that used GCF, saliva, and serum to correlate periodontal disease with clinical parameters (38, 44). One reason for this correlation is the abundance of MIP-1α in periodontally involved tissues. MIP-1α is predominantly localized within the connective tissue of the inflamed pocket epithelium and is responsible for immuno-modulatory mechanisms such as

mobilizing macrophages and initiating osteoclastic activity leading to bone resorption (45, 46). Moreover, the potential benefits of prophylactic antibiotics and antioxidants in promoting biocompatible and regenerative periodontal therapies for peri-implant tissues are that salivary levels of inflammatory and anti-inflammatory biomarkers may have implications for cardiovascular risk assessment in periodontitis patients with and without acute myocardial infarction.

Although salivary CRP and adiponectin offer a paradigm shift in isolation and localization of biomolecules in both health and disease, the ability to bypass inflammation *in situ* regardless of the condition and to stratify and correlate disease activity with a systemic condition is a challenge given the pathophysiological mechanisms that relate marginally close in terms of disease activity but abundantly in terms of ischemia as in myocardial infarction. We selected MIP-1α because of its discriminatory capacity and role in differentiating resident infection, characteristic of periodontitis, which was also reported in a study where the sensitivity of salivary MIP-1α was 90.3%. The authors investigated the role of MMP-8, 9, IL-1β, IL-6, and TNF-α along with MIP-1α and found that MIP-1α had the highest discriminatory role in periodontal disease among adults (47).

In this study, we found that salivary levels of these analytes changed with progressive periodontitis due to systemic inflammatory host response. We observed lower adiponectin levels in periodontitis and AMI patients, with considerably higher levels in controls and high levels of MIP-1α and CRP in periodontitis patients and higher levels in AMI patients with periodontitis. We believe that the abundant expression of CRP and MIP-1α in resident inflammatory conditions such as AMI and periodontitis could be due to aggravated periodontal breakdown, resulting in the expression of antimicrobial peptides due to a dysbiotic environment in the microbial dental film.

### 4.1 Limitations

While our study sheds light on the correlation between CRP and MIP-1α levels in periodontitis and AMI patients, some limitations inherent to cross-sectional studies restrict making predictive statements. For instance, although we observed elevated CRP levels in both AMI and periodontitis patients, whether high values are present post-MI or during the time leading to cardiac ischemia remains debatable. Moreover, our sample size is relatively small, which does not allow us to conclusively establish the association of these relevant biomarkers with MI or correlate it with resident periodontal infection.

Furthermore, the 48 h time frame for clinical evaluation in MI patients may not have provided enough statistical correlation to fully understand the presence of these biomarkers in all three groups during inflammatory conditions. Confounder variables may also have influenced our data, and their role cannot be ignored. Nonetheless, our findings can potentially inform future research and clinical practice.

## 5 Conclusions

The study attempted to explore the interrelationships between systemic anti-inflammatory and pro- inflammatory activities among biomarkers in AMI subjects. The findings suggest that salivary MIP-1α, CRP, and Adiponectin could potentially serve as a discriminatory tool for evaluating and assessing various stages of periodontal disease. However, the study's small sample size and cross-sectional nature limit the ability to draw predictive conclusions about increased levels of these biomarkers. Further longitudinal studies with increased sample sizes are warranted to validate these results and assess the sensitivity and specificity of these analytes in predicting periodontal activity and identifying individuals at risk for a cardiac event.

## Data Availability

The raw data supporting the conclusions of this article will be made available by the authors, without undue reservation.
